# Dissection of the *Caenorhabditis elegans* Microprocessor

**DOI:** 10.1093/nar/gkac1170

**Published:** 2023-01-04

**Authors:** Thuy Linh Nguyen, Trung Duc Nguyen, Minh Khoa Ngo, Tuan Anh Nguyen

**Affiliations:** Division of Life Science, The Hong Kong University of Science & Technology, Hong Kong, China; Division of Life Science, The Hong Kong University of Science & Technology, Hong Kong, China; Division of Life Science, The Hong Kong University of Science & Technology, Hong Kong, China; Division of Life Science, The Hong Kong University of Science & Technology, Hong Kong, China

## Abstract

Microprocessor (MP) is a complex involved in initiating the biogenesis of microRNAs (miRNAs) by cleaving primary microRNAs (pri-miRNAs). miRNAs are small single-stranded RNAs that play a key role in the post-transcriptional regulation of gene expression. Thus, understanding the molecular mechanism of MP is critical for interpreting the roles of miRNAs in normal cellular processes and during the onset of various diseases. MP comprises a ribonuclease enzyme, DROSHA, and a dimeric RNA-binding protein, which is called DGCR8 in humans and Pasha in *Caenorhabditis elegans*. DROSHA cleaves stem-loop structures located within pri-miRNAs to generate pre-miRNAs. Although the molecular mechanism of human MP (hMP; hDROSHA-DGCR8) is well understood, that of *Caenorhabditis elegans* MP (cMP; cDrosha-Pasha) is still largely unknown. Here, we reveal the molecular mechanism of cMP and show that it is distinct from that of hMP. We demonstrate that cDrosha and Pasha measure ∼16 and ∼25 bp along a pri-miRNA stem, respectively, and they work together to determine the site of cMP cleavage in pri-miRNAs. We also demonstrate the molecular basis for their substrate measurement. Thus, our findings reveal a previously unknown molecular mechanism of cMP; demonstrate the differences between the mechanisms of hMP and cMP; and provide a foundation for revealing the mechanisms regulating miRNA expression in different animal species.

## Graphical Abstract

**Graphical Abstract:**
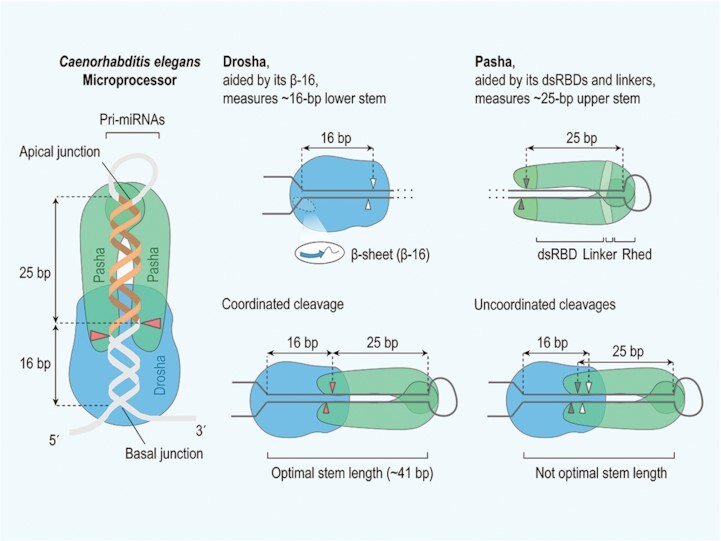
In the *Caenorhabditis elegans* Microprocessor complex, Drosha and Pasha subunits are coordinated to determine the cleavage sites of the complex in pri-miRNAs.

## INTRODUCTION

microRNAs (miRNAs) are small non-coding RNAs of ∼22 nucleotides (nt) in length that post-transcriptionally repress gene expression (1–3). In animals, most miRNAs are generated by an evolutionarily conserved pathway called miRNA biogenesis, which involves two RNase III enzymes, Microprocessor and DICER. Microprocessor (MP) is a complex located in the nucleus, which consists of DROSHA partnered with a double-stranded RNA-binding protein called DGCR8 in humans or Pasha in *Drosophila melanogaster* and *Caenorhabditis elegans*. The MP cleaves primary miRNAs (pri-miRNAs) to generate precursor miRNAs (pre-miRNAs). The resulting pre-miRNAs are transported to the cytoplasm, where DICER catalyzes the second cleavage to liberate miRNA duplexes of ∼22 bp (1,2). In *C. elegans*, this event is carried out by the human DICER homolog DCR-1 (4–6). These miRNA duplexes are loaded onto an Argonaute protein (AGO), which selects one of the two miRNA strands. In *C. elegans*, miRNAs are sorted into their AGO proteins, ALG-1, ALG-2 and ALG-5 (4,7–10). The resulting miRNA-AGO complex finds and silences the expression of the target mRNAs (1,2,11,12). The fidelity and efficiency of Microprocessor and DICER are critical for the maturation of miRNAs and, in turn, their cellular functions.

MP complexes were discovered in 2004 in many animals, including *C. elegans*, *D. melanogaster*, and humans (13–16). MPs are essential for miRNA biogenesis and normal cellular physiology in animals. For example, in human cells, depletion of DROSHA or DGCR8 results in a profound reduction of miRNA expression and cellular growth (17,18). In mice, DGCR8 knockout is embryonic lethal (at an early stage) (19). In *C. elegans*, MP-depleted embryos fail to undergo morphogenesis and organogenesis (20). Although MPs have been found in all animals, in the past two decades, the molecular mechanism of MP has only been extensively investigated in humans. Thus, the molecular mechanisms of MP in other animals, including *C. elegans*, are still largely unknown.

In the current understanding of the hMP mechanism, human DROSHA (hDROSHA) measures ∼13 bp from the basal junction of pri-miRNAs to the cleavage sites (21,22). The ∼13-bp measurement of hDROSHA requires its double-stranded RNA-binding domain (dsRBD) and the Belt helix in its central domain (23,24). hDROSHA also recognizes the UG and mGHG motifs located at the basal junction and in the lower stem of pri-miRNAs, respectively (21,25–27). midBMW, containing bulges, mismatches, or wobble bp in positions 10–12 from the cleavage sites, inhibits the unproductive cleavage of hDROSHA, and thus enhances its productive cleavage (28,29). Human DGCR8 (hDGCR8) binds to the apical loop and the UGU motif ∼22 bp away from the cleavage site (18,21,22,25,30,31). Although hDGCR8 neither measures the length of the stem nor determines the cleavage sites of hMP, it is essential for the hMP cleavage process in multiple ways. For example, it prevents unproductive cleavage by hDROSHA, which cleaves at the upper stem pri-miRNAs using apical junction recognition. hDGCR8 also increases the cleavage accuracy of hDROSHA by preventing alternative cleavages from occurring around the productive cleavage sites (21). Another motif, CNNC, is enriched in the 3p-segment of pri-miRNAs. This does not directly interact with hMP, but it binds to SRSF3, which recruits hDROSHA to the basal junction for more efficient and accurate cleavage (25,32,33). While CNNC and various other motifs (i.e. UG, mGHG, UGU) are known to be enriched in human pri-miRNAs and conserved in the pri-miRNAs of many organisms from flies to humans, UG, UGU and CNNC are not enriched or conserved in *C. elegans* pri-miRNAs (25,26,34). In contrast, midBMW are found in pri-miRNAs from many organisms, including humans and *C. elegans* (28,29).

Discovered at the same time as hMP, *C. elegans* Microprocessor (cMP) is composed of *C. elegans* Drosha (cDrosha) and Pasha, a homolog of hDGCR8 (13). The domain structures and polypeptide sequences of cMP and hMP are similar (i.e. according to the BLAST comparison function, hDROSHA and cDrosha share 36% polypeptide identity, and DGCR8 and Pasha share 25% polypeptide identity). However, when *C. elegans* pri-miRNAs (cel-pri-miRNAs) were ectopically expressed in human cells, they were not cleaved efficiently by hMP (25,34). This is because hMP requires multiple structures and sequencing motifs (i.e. UG, UGU, and CNNC, absent in cel-pri-miRNAs) to precisely and efficiently cleave pri-miRNAs. This suggests that cMP and hMP might utilize distinct substrate interaction approaches and cleavage mechanisms. Here, we revealed the molecular mechanism of cMP by conducting high-throughput (HT) cleavage assays for all the annotated cel-pri-miRNAs with cMP. In addition, we dissected the roles of cDrosha and Pasha in determining the cleavage sites of cMP; explained their substrate measurement; and provided a mechanistic explanation for the alternative cleavages.

## MATERIALS AND METHODS

### Plasmid construction

The pXab-cel-Drosha and pXG-cel-Pasha (PASH-1) plasmids were constructed as previously described (35). The mutant protein-encoding plasmids were generated from these 2 original plasmids using either the In-Fusion cloning or the T4 DNA ligation method. The cloning primers and methods are listed in Supplementary Table S1.

### Recombinant protein purification

The purification of cMP wild-type (WT) or mutant was performed as previously described (35). The cDrosha was prepared as the cDrosha-NLScPa2 complex, in which NLS is a nuclear localization sequence (PKKKRKV), and cPa2 is the fragment of Pasha (amino acids 595–751). The purification of the cDrosha-NLScPa2 complex was performed in a similar way to that of cMP (35). The hDROSHA was prepared as the D3-G2 complex (28). The hMP was prepared as the NLSD3-DGCR8 complex (36). D3 and G2 are the fragments of DROSHA (amino acids 390–1365) and DGCR8 (amino acids 701–773), respectively. NLS is a nuclear localization sequence (PKKKRKV).

### *C. elegans* pri-miRNA substrate preparation

Each DNA sequence coding for pri-miRNAs contained a DNA sequence coding for pre-miRNA and its 30 bp upstream and 25 bp downstream sequence in the *C.elegans* genomic DNA. Each pri-miRNA-coding DNA was added with a T7 promoter sequence at its 5p-end to generate a DNA template for *in vitro* transcription (IVT), so-called IVT-DNAs. The IVT-DNAs were synthesized by 2 methods. Twenty IVT-DNAs were produced by Phusion™ Hot Start II DNA Polymerase (Thermo Scientific) in the PCR reaction using a pair of primers and *C.elegans* genomic DNA or a synthetic ssDNA backbone as PCR template. One hundred and seventeen IVT-DNAs were made by Klenow Fragment (Thermo Scientific), which extended the partial dsDNAs generated from annealing pairs of synthetic ssDNAs. The primer sequences and synthesis methods for each IVT-DNA are shown in Supplementary Table S2. The pri-miRNAs were synthesized in a 20 μl IVT reaction mixture containing 200 ng IVT-DNA using the MEGAscript T7 Kit (Invitrogen). The IVT mixture was incubated at 37°C for 12 h. The IVT-DNA templates were then digested using TURBO DNase (Thermo Scientific). The reaction mixture was treated with 20 μl 2x TBE-Urea buffer and denatured at 75°C for 5 min. The denatured RNA was loaded onto a pre-run 10% Urea-PAGE. The RNA at the expected size was gel-purified and air-dried. Finally, the RNA was dissolved in distilled water and stored at -80°C for later use.

### *In vitro* pri-miRNA cleavage assay

The pri-miRNA cleavage assay was performed in 10 μl of a buffer containing 50 mM Tris–HCl (pH 7.5), 150 mM NaCl, 10% glycerol, 0.2 μg/μl BSA, 1 mM DTT and 2 mM MgCl_2_. Each cleavage reaction contains 4 pmol of RNA substrate. We used 0.4 pmol of *C. elegans* Drosha (cDrosha), 0.2 pmol of *C. elegans* Microprocessor (cMP) (WT or mutant), 12 pmol of human DROSHA (hDROSHA, D3-G2), and 2 pmol of human Microprocessor (hMP, NLSD3-DGCR8) (otherwise stated in the figure legends) in one cleavage reaction. The human protein amounts were estimated by Bradford protein assays. The *C. elegans* proteins were estimated by comparing them with the standard proteins (BSA) in the SDS-PAGE. The reaction was incubated at 37°C for 2 h. After that, the reaction was terminated by adding 10 μl of 2× TBE–urea sample buffer, immediately chilled on ice, and added with 20 μg proteinase K (Thermo Fisher). The incubation was kept at 37°C for 15 min, followed by 50°C for 15 min, and finally at 95°C for 5 min. The cleavage mixture was resolved on a pre-run 12% urea-PAGE in 1× TBE buffer, and the gel was run at 300 V for 50 min. The gel was stained with SYBR™ Green II RNA Gel Stain (Invitrogen) for 10 min and imaged by the Bio-Rad Gel Doc XR+ system. The RNA band intensities were measured using Image Lab 5.0. The cleavage sites of enzymes in the cleavage assays were determined by the F2 sequencing or referred from the HT cleavage assays in this study, the lengths of cleaved products in the gels, and previous reports (28,36).

### High-throughput cleavage library preparation

We collected 145 *C. elegans* pri-miRNAs from MirGeneDB v2.0 (37) and discarded seven duplicated pri-miRNAs sharing the same miRBase ID (38). Cel-pri-mir-229 is also excluded since its length is longer than 150 nt. As a result, 137 pri-miRNAs were selected and synthesized to generate a *C. elegans* pri-miRNA pool. We used 12 pmol of cel-pri-miRNA pool and 2.4 and 1.2 pmol of cDrosha and cMP, respectively. The HT pri-miRNA cleavage reaction was then carried out similarly as described above.

The cleaved RNA products (50–70 nt) from the HT cel-pri-miRNA cleavage reactions were gel-purified and then dissolved in 10 μl distilled water. An equal amount of hsa-pre-mir-16-1, obtained from the cleavage of hsa-pri-mir-16–1 by hMP, was mixed into the RNA products from the cleavage of each enzyme to serve as a cloning control. The resulting RNA mixture was ligated to 10 pmol of 3′-adapter (4N-RA3, /5rApp/NN NNT GGA ATT CTC GGG TGC CAA GG/3ddC/) in a 20 μl reaction mixture containing 1 μl T4 RNA Ligase 2, truncated KQ (NEB, M0373L), 2 μl 10× T4 RNA ligase buffer, 6 μl of 50% PEG8000, and 0.5 μl of SUPERase•In RNase inhibitor (20 U/μl). The mixture was incubated at 25°C for 16 h. After that, it was treated with 20 μl 2× TBE–urea sample buffer, denatured at 75°C for 5 min, and loaded onto a pre-run 12% urea-PAGE. The RA3-ligated RNAs were gel-purified and dissolved in distilled water. The dissolved RA3-ligated RNAs were ligated to 10 pmol of 5′-adapter (RA5-4N, GUU CAG AGU UCU ACA GUC CGA CGA UCN NNN) in a reaction mixture containing 1 μl T4 RNA ligase I (NEB, M0204L), 2 μl 10× T4 RNA ligase buffer, 6 μl of 50% PEG8000, and 0.5 μl of SUPERase•In RNase inhibitor (20 U/μl). The reaction was kept at 25°C for 16 h. The RA5/RA3-ligated RNAs were reverse transcribed with R-RA3 primer (TTG GCA CCC GAG AAT TCC A) using Superscript IV reverse transcriptase (Invitrogen, 18090050). The resulting cDNA was PCR-amplified using a pair of sequencing primers RP1 (AAT GAT ACG GCG ACC ACC GAG ATC TAC ACG TTC AGA GTT CTA CAG TCC GA) and one of RPIx.

Ten pmol of the pri-miRNA pool were ligated with 30 pmol of 3′-adapter, similarly as described above for the cleaved RNAs. The RA3-ligated RNAs were reverse transcribed with the cirRTP primer (/5Phos/NNN NNN GA TCG TCG GAC TGT AGA ACT CTG AAC /iSp18/CC TTG GCA CCC GAG AAT TCC A) using Superscript IV reverse transcriptase (Invitrogen, 18090050). The cDNA was treated with 0.1 mM NaOH at 95°C for 5 min to eliminate the RNAs and gel-purified. The purified cDNAs were circularized using the CircLigase™ ss-DNA Ligase (Epicentre, CL4115K) and loaded onto a pre-run 18% urea-PAGE. The circular DNAs were gel-purified and PCR-amplified using sequencing primers RP1 (AAT GAT ACG GCG ACC ACC GAG ATC TAC ACG TTC AGA GTT CTA CAG TCC GA) and one of RPIx.

The DNA libraries were sequenced with Illumina NovaSeq 6000 system in 150 bp paired-end mode. Sequencing data were deposited in Gene Expression Omnibus (GEO) with the accession number GSE212229 (reviewer access: kferoaumvzyzheh).

### High-throughput cleavage library processing and analysis

First, cutadapt was used to remove adapters from both ends of raw reads (-a TGGAATTCTCGGGTGCCAAGG -A GATCGTCGGACTGTAGAACTCTGAAC) (39). Next, fastq-join were applied to join paired-end reads (-p 5) (40). The low-quality joined reads and duplicated reads were removed using fastq_quality_filter (-q 20 -p 90) and fastx_collapser, respectively (http://hannonlab.cshl.edu/fastx_toolkit/index.html). Then, the randomized barcodes at both ends of the remaining reads were trimmed by cutadapt (-u 4 -u -6: 4 nt in 5p-end and 6 nt in 3p-end for reads in control samples, -u 4 -u -4: 4 nt at both ends for reads in product samples) (39). Next, bowtie2 was used to map the trimmed reads the build-in reference which contains the sequences of 137 *C. elegans* pri-miRNAs (–local –norc -p 4 -k 5) (41). The pri-miRNA sequences contain 30- and 25-nt extensions from 5p- and 3p-ends of pre-miRNA sequences, respectively. Additionally, GGG also was appended to the 5p-end of pri-miRNA reference sequences to represent the genuinely pri-miRNAs generated from IVT. The unique mapped reads were collected. The 132 out of 137 pri-miRNAs with more than 20 read counts in the control sample were taken for further analysis. For the control sample, the raw counts were normalized as counts per million. For cleaved product samples, the raw counts were normalized by that of hsa-pre-miR-16-1, serving as an internal control of the cleaved products in the cloning process. Then, normalized counts were multiplied with a scaling factor of 1,000 to make the normalized counts higher than 0.1.

We assigned 5p and 3p-ends of annotated pre-miRNAs in MirGeneDB as CL0. The product reads, whose 5p or 3p-ends ranged from CL-5 to CL5, were selected for further analysis. Let P be the position ranging from CL-5 to CL5 on the 5p-strand of pri-miRNAs, N_P_ be the normalized counts of the RNA products cleaved at position P; N_S_ be the normalized counts of the pri-miRNA in the control sample, and 0.1 be a pseudocount. The global cleavage efficiency score was calculated as log_2_(∑*N*_P_ + 0.1) – log_2_(*N*_S_ + 0.1). The cleavage efficiency score at position P was calculated as log_2_(*N*_P_ + 0.1) – log_2_(*N*_S_ + 0.1). The cleavage accuracy score at position P was calculated as *N*_P_/∑*N*_P_.

The cleavage site changes between each cMP mutant and its WT were quantified using the Kullback–Leibler divergence (42) that compared the distributions of cleavage accuracy scores from CL-5 to CL5 on the 5p-strand.

### Secondary structure analysis of pri-miRNAs

We collected 10,895 pri-miRNA sequences (20-nt extension at both ends of pre-miRNA sequences) from 45 species in MirGeneDB v2.0 (37). We removed duplicated pri-miRNAs with similar miRBase IDs (mirbase.org (38)). Secondary structures of pri-miRNAs were predicted using RNAfold (43). The pri-miRNAs with multiple loops were discarded. The fraction of mismatches at different positions along the pri-miRNA stems was determined for the 5p-strand only.

The basal and apical junctions were determined where the stems were separated into single-stranded regions with at least 3 mismatches from position -10 to -25 and from position +21 to +30 on 5p-strands of pri-miRNAs, respectively. The 5p-end of pre-miRNAs was assigned as position 0. The distances of cleavage sites to basal and apical junctions were estimated as the number of bp between them.

The UG and UGU/GUG motifs were examined between positions –14 to –12 and +21 to +26 on the 5p-strands of pri-miRNAs, respectively. The midBMW pri-miRNAs contained the unmatched or wobble bp between positions +10 to +12 on the 5p-strands of pri-miRNAs. The CNNC motif was scanned from positions –16 to –21 on the 3p-strands of pri-miRNAs. mGHG was investigated from positions –5 to –3 on the 3p-strands of pri-miRNAs. Noted that pri-miRNAs containing any bulges between positions –5 to –3 on the 3p-strands were excluded from mGHG investigation. The mGHG pri-miRNAs are those pri-miRNAs containing the mGHG motif with the mGHG score >38 (27).

## RESULTS

### High-throughput *C. elegans* pri-miRNA cleavage assays for cDrosha and cMP

**Figure 1.**
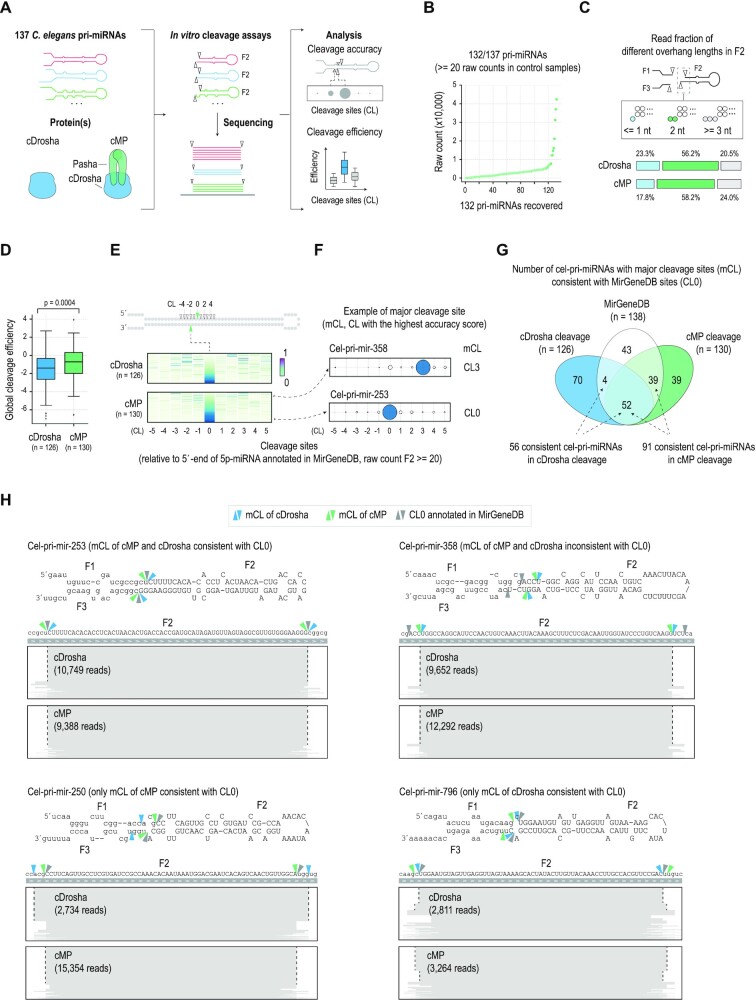
High-throughput cel-pri-miRNA cleavage assays. (**A**) Schematic diagram illustrating the layout of the high-throughput (HT) cel-pri-miRNA cleavage assay. (**B**) The graph shows that in the HT cleavage assays, 132 out of 137 cel-pri-miRNAs were identified in the control (uncleaved cel-pri-miRNA) sample. (**C**) The fraction of cleaved RNAs (F2 fragments) containing different overhang nt. (**D**) The global cleavage efficiency of cDrosha and cMP was estimated as the ratio of the products cleaved at all cleavage sites to the original substrate. (**E**) The cleavage site patterns of cel-pri-miRNAs. The upper panel shows the diagram of pri-miRNA and the cleavage sites (CL) at different positions. CL0 indicates the canonical cleavage sites of cel-pri-miRNAs annotated in MirGeneDB (37). CL1 to CL5 and CL-1 to CL-5 are the cleavage sites located 1–5 nt downstream and upstream of CL0, respectively, on the 5p-strand of cel-pri-miRNAs. The middle and lower panels show the cleavage accuracy scores of cel-pri-miRNAs by cDrosha (middle panel) and cMP (lower panel) at different cleavage sites. Each line indicates one cel-pri-miRNA. (**F**) The cleavage sites of cMP in cel-pri-mir-358 and cel-pri-mir-253. The blue circles indicate the major cleavage site (mCL), the cleavage site with the highest cleavage accuracy score. (**G**) The number of cel-pri-miRNAs containing mCLs, consistent with their annotated cleavage sites (CL0). (**H**) Cleavage patterns of representative cel-pri-miRNAs in each group in panel (G). The blue, green, and gray arrowheads indicate the major cleavage sites (mCL) of cDrosha, cMP, and CL0 annotated in MirGeneDB, respectively.

The cDrosha and cMP complexes were prepared as described in the Materials and Methods (Supplementary Figure S1A, B). The cleavage activity of cDrosha and cMP was preliminarily assessed with four cel-pri-miRNAs (Supplementary Figure S1C, D), after which HT cleavage assays were conducted for cDrosha and cMP (cDrosha-Pasha) with all the 137 cel-pri-miRNAs listed in the MirGeneDB v2.0 (37). These cel-pri-miRNAs and their cleaved products (F2, pre-miRNAs) were cloned and sequenced using NGS (Figure 1A, Supplementary Figure S1E). After sequencing, 132/137 cel-pri-miRNAs, which contained >=20 raw counts in the control samples (i.e. the pri-miRNA sample), were identified (Figure 1B). Most of the F2 fragments resulting from the cDrosha or cMP cleavage contained 2 nt overhanging at their 3p-ends, indicating that they were typical products of RNase III enzymes (Figure 1C). In addition, the cleavage efficiency of cMP was higher than that of cDrosha, indicating the stimulatory effect of Pasha on the cleavage activity of cDrosha (Figure 1D). Consistent with findings in hMP (28,29), cDrosha and cMP exhibited higher cleavage efficiency in cel-pri-miRNAs containing midBMW (Supplementary Figure S1F).

We determined the cleavage sites of cDrosha and cMP in each cel-pri-miRNA from the HT cleavage assays. The cleavage sites are presented as CLx, such that the ‘x’ indicates the position of the cleavage site (from –5 to 5) on the 5p-strand of the cel-pri-miRNA (Figure 1E). The annotated cleavage sites from the MirGeneDB are shown as CL0 (Figure 1E, F). Since cDrosha and cMP cleaved most of the cel-pri-miRNAs at multiple sites (Supplementary Table S3), we quantified the cleavage frequency of all the cleavage sites of the enzymes for each cel-pri-miRNA and identified the major cleavage site (mCL); i.e. the site with the highest frequency (Supplementary Figure S1G). We found that 91/130 cel-pri-miRNAs were cleaved by cMP at the MirGeneDB-annotated sites (CL0), whereas 39/130 were cleaved more frequently at sites other than CL0 (Figure 1G). In addition, cMP and cDrosha cleaved 52 cel-pri-miRNAs at the CL0, but they exhibited different mCLs in another 43 cel-pri-miRNAs. These data indicate that Pasha works together with cDrosha to determine the cMP cleavage sites (Figure 1G, H).

### cDrosha measures ∼16 bp of the lower stem

**Figure 2.**
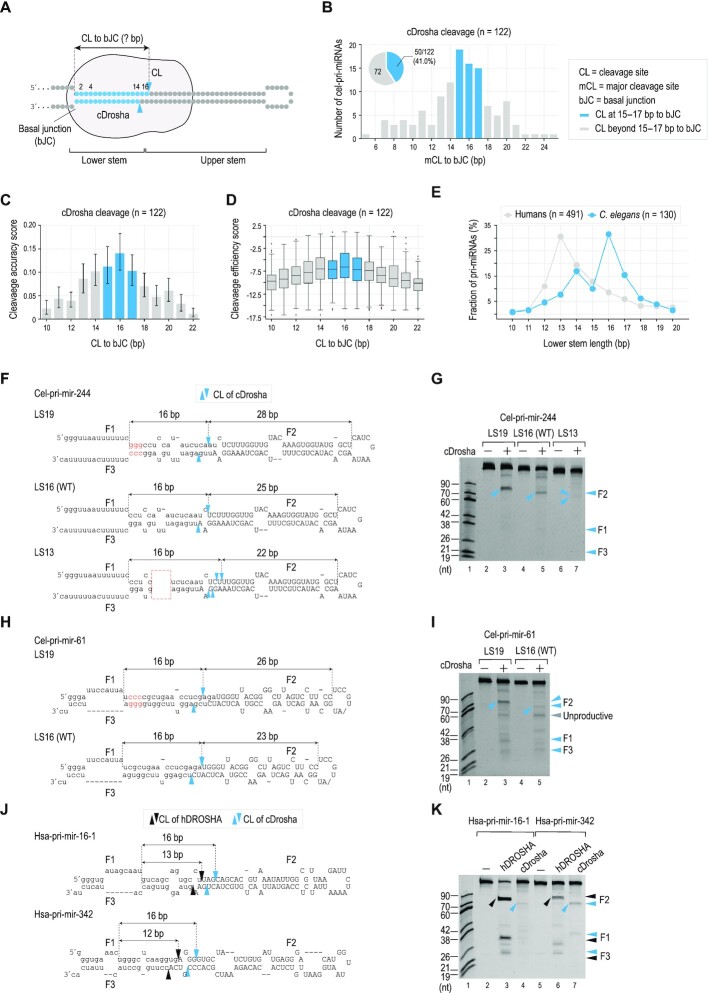
cDrosha measures ∼16 bp of the lower stem. (**A**) The schematic diagram shows that cDrosha measures the length of lower stems in cel-pri-miRNAs to determine its cleavage sites. (**B**) The number of cel-pri-miRNAs containing different distances between their mCLs of cDrosha and the basal junction (bJC). The pie chart shows the fraction of cel-pri-miRNAs containing the 15–17-bp mCL-bJC distance. 122/126 cel-pri-miRNAs cleaved by cDrosha were selected for this analysis. The remaining four cel-pri-miRNAs (cel-pri-mir-240, cel-pri-mir-4808, cel-pri-mir-84 and cel-pri-mir-246) were excluded as their CL0 was not located in their stems. (**C, D**) The average (**C**) cleavage accuracy and (**D**) efficiency scores of the CLs of cDrosha at different distances from the bJC. (**E**) Graph showing the percentage of pri-miRNAs containing different lengths of the lower stem in *C. elegans* and humans. 130/138 cel-pri-miRNAs and 491/508 hsa-pri-miRNAs were selected for this analysis. The remaining 8 cel-pri-miRNAs and 17 hsa-pri-miRNAs were excluded as they contained multiple-loop structures. (**F**, **H**, **J**) Diagrams and sequences of cel-pri-mir-244 (**F**), cel-pri-mir-61 (**H**), hsa-pri-mir-16-1 and hsa-pri-mir-342 (**J**). The inserted nt are red letters. The deleted nt are shown in the red dashed box. (**G**, **I**) Gels showing the results from the *in vitro* cleavage assays of cDrosha for cel-pri-mir-244, cel-pri-mir-61, and their variants. The F2 products of the cleavage assay in (**I**) were confirmed by sequencing (Supplementary Table S4). (**K**) Gel showing the results from the *in vitro* cleavage assays of cDrosha and hDROSHA for hsa-pri-mir-16-1 and hsa-pri-mir-342. In all panels of Figure 2, the blue and black arrowheads indicate the cleavage sites of cDrosha and hDROSHA, respectively. The gray arrowhead indicates the unproductive cleavage of cDrosha.

We determined mCL of cDrosha in 122 out of 126 cel-pri-miRNAs, identified in the HT cleavage assays. Four cel-pri-miRNAs, cel-pri-mir-240, cel-pri-mir-4808, cel-pri-mir-84 and cel-pri-mir-246, were excluded from this analysis because their CL0 was beyond their stems. We found that cDrosha cleaved around 41% pri-miRNAs at 15–17 bp from the basal junction (Figure 2A, B). In addition, cDrosha also achieved higher cleavage accuracy and efficiency scores when cleaving cel-pri-miRNAs at these positions (Figure 2C, D). These observations suggest that cDrosha measures 15–17 bp from the basal junction to the cleavage sites in cel-pri-miRNAs, which is different from the well-demonstrated 13-bp measurement of hDROSHA.

We then analyzed human pri-miRNAs (hsa-pri-miRNAs) and cel-pri-miRNAs and consistently found that most human pri-miRNAs contain a lower stem of ∼13 bp, whereas many cel-pri-miRNAs possess a longer lower stem, i.e. ∼15–17 bp (Materials and Methods, Figure 2E). These results suggest that many cel-pri-miRNAs adopt a lower stem of 15–17 bp for cDrosha to measure.

To confirm the 16-bp (short of 15–17 bp) measuring mechanism of cDrosha, we conducted cleavage assays for cDrosha and two cel-pri-miRNAs (cel-pri-mir-244 and cel-pri-mir-61) and found that cDrosha alone cleaved these cel-pri-miRNAs at 16 bp from their basal junctions (Figure 2F–I). We then elongated the lower stem of these two cel-pri-miRNAs by 3 bp to generate LS19 variants and observed that in each case, the cleavage sites of cDrosha were shifted 3 bp toward the basal junction, keeping a 16-bp constant distance between the basal junction and its cleavage sites (Figure 2F–I). Of note, cDrosha showed an enhanced cleavage efficiency for cel-pri-mir-244 LS19 when compared with the LS16 variant (Figure 2G, compare lanes 3 and 5) because the mismatch (C-A) in position –1 of LS16 was inhibitory to cDrosha cleavage (28,29). In addition, we shortened the lower stem of cel-pri-mir-244 to generate an LS13 variant and showed that cDrosha shifted its cleavage sites 3 bp away from the basal junction to maintain a ∼16-bp constant distance between the basal junction and its cleavage sites (Figure 2F, G). These results demonstrate the difference between cDrosha and hDROSHA, with the former measuring ∼16 bp from the basal junction of the stem structure and the latter having a 13-bp measuring mechanism.

To observe the difference in the substrate measuring mechanisms between cDrosha and hDROSHA, we cleaved four human pri-miRNAs (hsa-pri-miRNAs), hsa-pri-mir-16–1, hsa-pri-mir-342, hsa-pri-mir-31, and hsa-pri-mir-125a with cDrosha and hDROSHA (Figure 2J, K, Supplementary Figure S2A–D). As previously established mechanisms (3,21,22,26,27,33), hDROSHA showed the expected cleavage sites at ∼13 bp from the basal junction. Interestingly, the cleavage of cDrosha resulted in shorter F2 fragments, which were ∼16 bp from the basal junctions of these hsa-pri-miRNAs (Figure 2J, K, Supplementary Figure S2C, D). These data further demonstrate the 16-bp measuring mechanism of cDrosha and highlight the distinct substrate-measuring methods of cDrosha and hDROSHA.

### Pasha measures ∼25 bp of the upper stem

**Figure 3.**
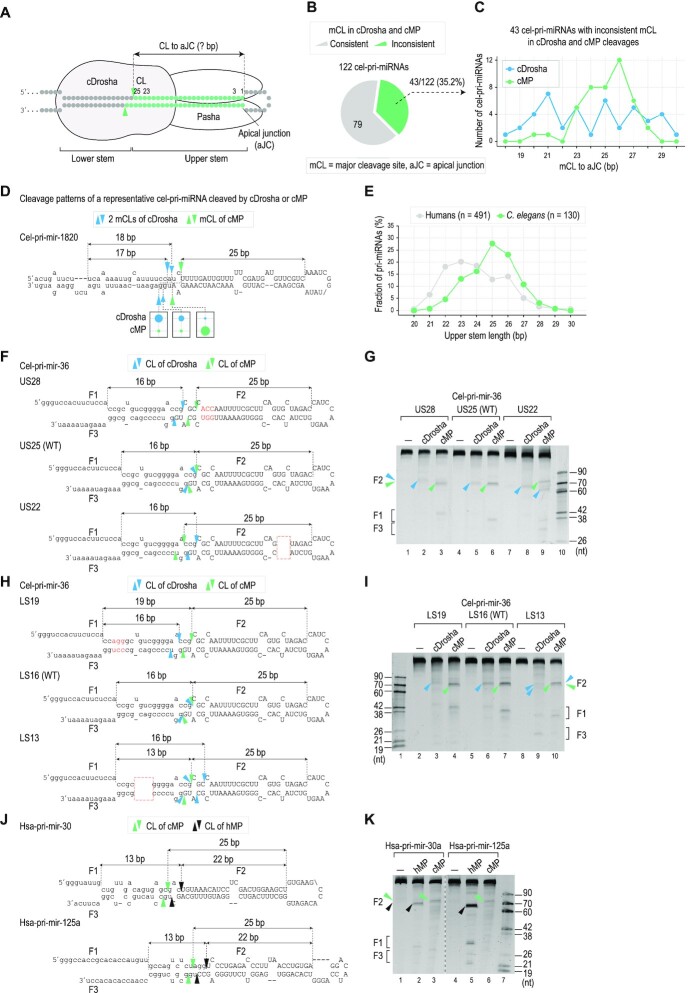
Pasha measures ∼25 bp of the upper stem. (**A**) The schematic diagram shows that Pasha measures the lengths of the upper stems in cel-pri-miRNAs and determines the cleavage sites of the cMP complex. (**B**) The fraction of cel-pri-miRNAs containing the different major cleavage sites (mCLs) between cDrosha and cMP, so-called inconsistent mCLs. (**C**) The number of cel-pri-miRNAs containing inconsistent mCLs at different distances to the apical junction (aJC). (**D**) The cleavage patterns of cDrosha and cMP in a representative cel-pri-miRNA, cel-pri-mir-1820. (**E**) Graph showing the percentage of pri-miRNAs containing different lengths of the upper stem in *C. elegans* and human pri-miRNAs. 130/138 cel-pri-miRNAs and 491/508 hsa-pri-miRNAs were selected for this analysis; the remaining were excluded as they contained multiple-loop structures. (**F**, **H**, **J**) Diagrams and sequences of (**F, H**) cel-pri-mir-36 and its variants and (**J**) hsa-pri-mir-30a and hsa-pri-mir-125a. The inserted nt are red letters. The deleted nt are shown in the red dashed box. (**G, I**) Gels showing the results from the *in vitro* cleavage assays of cDrosha and cMP. (**K**) Gel showing the results from the *in vitro* cleavage assays of cMP and hMP. In all panels of Figure 3, the blue and green arrowheads indicate the cleavage sites of cMP, determined by cDrosha and Pasha, respectively. The black arrowheads indicate the cleavage sites of the human Microprocessor, hMP.

To identify the function of Pasha in determining the cMP cleavage site, we compared the distance from the cleavage sites of cMP and cDrosha to the apical junctions in 122 cel-pri-miRNAs (Figure 3A). We found that 79/122 cel-pri-miRNAs were consistently cleaved by cMP and cDrosha at the same positions. This indicates that cDrosha plays a significant role in determining the cleavage sites of cMP in these cel-pri-miRNAs (Figure 3B). However, 43/122 cel-pri-miRNAs were cleaved differently with cMP and cDrosha, suggesting the influence of Pasha in the cleavage site determination of cMP (Figure 3B). cDrosha cleaved these 43 cel-pri-miRNAs (i.e. termed the ‘inconsistent’ group in Figure 3B) at various positions from the apical junction (Figure 3C, D), which were primarily different from the annotated cleavage sites of cMP (Supplementary Figure S3A). Unlike cDrosha, cMP cleaved most of these 43 cel-pri-miRNAs at 24–26 bp from the apical junction (Figure 3C, D) and these cMP-cleaved positions mainly coincided with the annotated cleavage sites of cMP (Supplementary Figure S3A). In addition, the cleavage sites of cMP in 79 cel-pri-miRNAs, cleaved consistently between cDrosha and cMP, were also mainly at 24–26 bp from the apical junction (Supplementary Figure S3B). Furthermore, cMP cleaved all the 122 cel-pri-miRNAs with higher cleavage efficiency and accuracy scores at 24–26 bp from the apical junction (Supplementary Figure S3C, D). These results indicate that Pasha measures 24–26 bp from the apical junction of the upper stem and thus helps to determine the site of cleavage by cMP.

We then compared the upper stems of pri-miRNAs from *C. elegans* with those from humans. Consistent with previous studies (26,44,45), the upper stems of most human pri-miRNAs are between 22 and 24 bp (Figure 3E). However, the upper stems of cel-pri-miRNAs are slightly longer, i.e. between 24 and 26 bp (Figure 3E). This feature of cel-pri-miRNAs is suitable for the 24–26-bp measurement of Pasha.

To confirm the 25-bp (short of ∼24–26 bp) measurement of Pasha, we changed the upper stem length (25 bp) of cel-pri-mir-36 by inserting or deleting 3 bp (Figure 3F). We found that cDrosha cleaved these cel-pri-miRNA variants at 16 bp from the basal junctions, resulting in F2 fragments of different lengths (Figure 3G, lanes 2, 5 and 8). However, when in complex with Pasha, cDrosha cleaved three cel-pri-mir-36 variants at a constant distance (25 bp) from the apical junction, resulting in same-length pre-miRNAs (F2 products) (Figure 3G, lanes 3, 6 and 9). This indicates that Pasha moved cDrosha from its initially-determined place (i.e. 16 bp from the basal junction) to the new location (i.e. 25 bp from the apical junction) in the two mutated variants of cel-pri-mir-36. In addition, we tested cDrosha and cMP with the cel-pri-mir-36 variants containing different lower stem lengths (LS19, LS16, and LS13). Again, we found that cDrosha alone cleaved the cel-pri-mir-36 LS19 and LS16 variants using the 16-bp measurement, which resulted in F2 products of different lengths (Figure 3H, I, lanes 3 and 6). Notably, cDrosha cleaved the LS13 variant at a new position, 16 bp from the basal junction, but still retained some activity at the original position (Figure 3I, lane 9). However, cMP cleaved all three variants at the exact location, 25 bp from the apical junction, generating same-length F2 fragments (Figure 3H, I, lanes 4, 7 and 10).

Furthermore, cMP also cleaved hsa-pri-mir-30a and hsa-pri-mir-125a at 25 bp from the apical junction. These cMP cleavage sites differed from those of hMP as they were ∼13 bp and ∼22 bp from the basal and apical junctions, respectively (Figure 3J, K). These results demonstrate that Pasha measures 25-bp from the apical junction of the upper stem of cel-pri-miRNAs, once again showing that it determines the site of cleavage by cMP.

### The coordination of two measuring mechanisms for optimal cleavage activity of cMP

**Figure 4.**
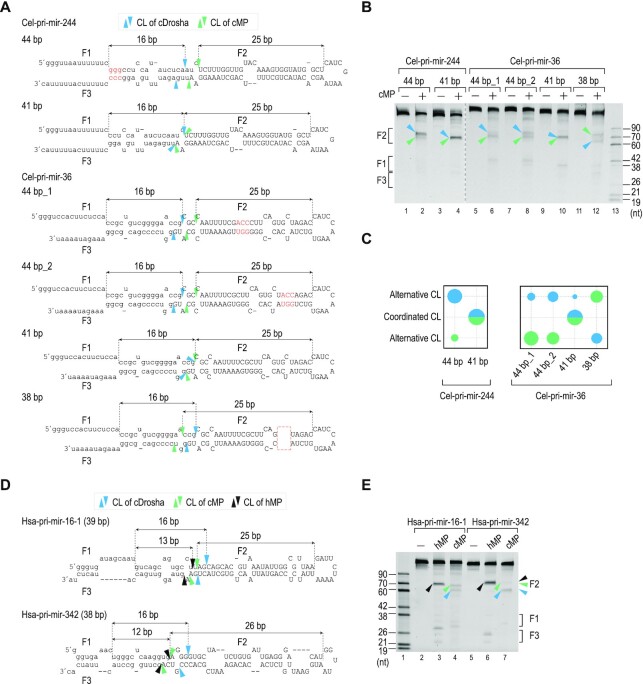
The coordination of cDrosha and Pasha in determining the cleavage sites. (**A**, **D**) The diagrams and sequences of (**A**) cel-pri-mir-244, cel-pri-mir-36, and their variants, and (**D**) hsa-pri-mir-16–1 and hsa-pri-mir-342. The inserted nt are red letters. The deleted nt are shown in the red dashed box. (**B, E**) Gels showing the results of the *in vitro* cleavage assays of cMP and hMP. (**C**) The quantitative plots of the cleavage assays were conducted in triplicate, shown in (**B**). In all panels of Figure 4, the blue and green arrowheads indicate the cleavage sites of cMP, determined by cDrosha and Pasha, respectively. The black arrowheads indicate the cleavage sites of the human Microprocessor, hMP. The sizes of the circles indicate the relative cleavage efficiencies.

Since cDrosha and Pasha measure ∼16-bp and ∼25-bp of the stem, respectively, they might coordinate the cleavage of cel-pri-miRNAs containing ∼41-bp stem length accurately. In addition, altering the stem length to be longer or shorter than ∼41-bp might affect their cooperation, resulting in alternative cleavages. We showed that cMP cleaved 41-bp cel-pri-miRNAs, including cel-pri-mir-244_41bp (WT) and cel-pri-mir-36_41bp (WT), at a single cleavage site (Figure 4A–C). However, cMP cleaved longer cel-pri-miRNAs, including cel-pri-mir-244_44bp (44 bp), cel-pri-mir-36_44bp_1 (44 bp), cel-pri-mir-36_44bp_2 (44 bp), and the shorter cel-pri-miRNA, cel-pri-mir-36_38bp (38 bp), at two separate cleavage sites, which are 16-bp and 25-bp from the basal and apical junctions, respectively (Figure 4B, C). In addition, we identified in the HT cleavage assays two cel-pri-miRNAs, containing 38-bp and 40-bp stems, which were cleaved at alternative sites, using both cDrosha and Pasha measurements (Supplementary Figure S4A–C).

In addition, we showed that cMP cleaved hsa-pri-mir-16-1 (39 bp) and hsa-pri-mir-342 (38 bp) at two different positions using both cDrosha and Pasha measurements (Figure 4D, E). Furthermore, we demonstrated that cDrosha and cMP cleaved the 38-bp artificial pri-miRNA (AS-1) at two different positions using two separate measuring mechanisms, the 16-bp measurement of cDrosha in cDrosha alone and the 25-bp measurement of Pasha in cMP. However, cDrosha and cMP cleaved the 41-bp artificial pri-miRNA (AS-2) at similar positions when the two cDrosha and Pasha measurements agreed at the same position (Supplementary Figure S4D, E). These results again confirm the 16-bp and 25-bp measuring mechanisms of cDrosha and Pasha, respectively, and demonstrate the cooperation of both these subunits to determine the site of cleavage by cMP.

### Structural basis of cDrosha measurement

**Figure 5.**
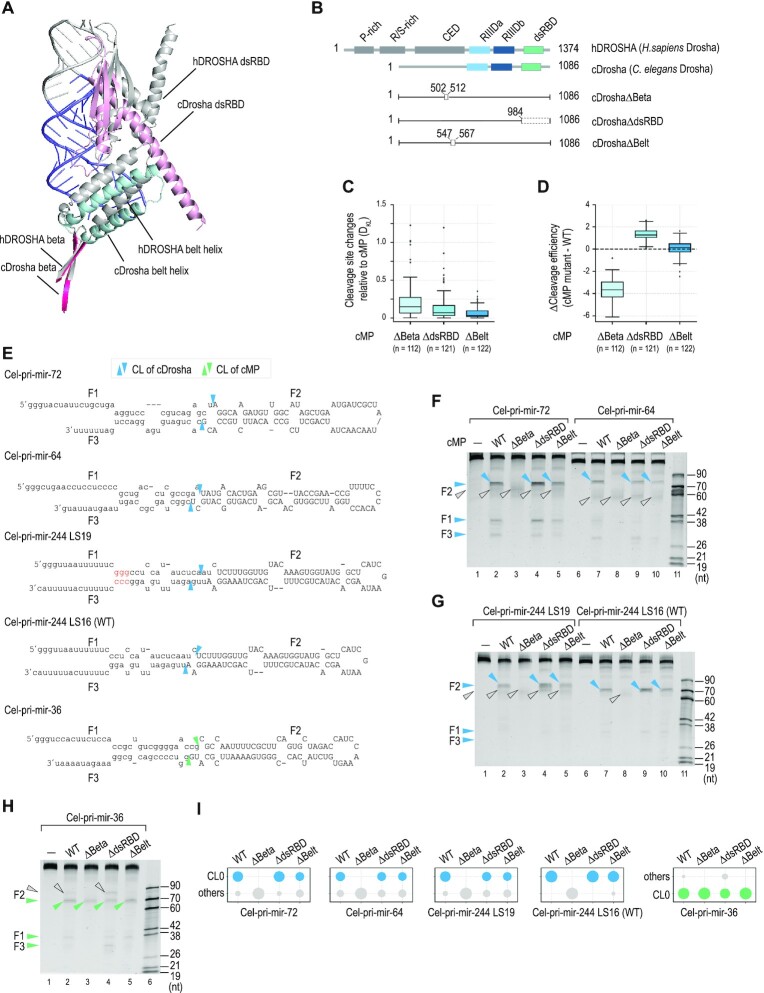
Structural basis of cDrosha measurement. (**A**) The 3D structure of cDrosha predicted by Alphafold2 (46,47) was superimposed onto that of hDROSHA in complex with RNA (PDB: 6V5B, or a similar model in PDB: 6LXD). The lower stem of the RNA is shown in slate, and the dsRBD, Belt helix, and β-sheet of cDrosha are shown in light pink, pale cyan, and hot pink, respectively. (**B**) The protein domains of hDROSHA and cDrosha. The numbers indicate the positions of the amino acids in the polypeptides, and the white rectangles show the deleted regions of the mutant cDrosha. (**C**) Mutations in the β-sheet changed the cleavage sites of cDrosha in the HT cleavage assays. When evaluating mutant and WT cDrosha, changes in the cleavage site were calculated using the Kullback-Leibler divergence. This was used to compare the distributions of cleavage accuracy scores from CL-5 to CL5 on the 5p-strand. (**D**) Mutations in the β-sheet reduced the relative cleavage efficiency scores of cDrosha. The cleavage efficiency scores of mutant cDrosha for each cel-pri-miRNA were calculated, as described for WT cDrosha. (**E**) Diagrams and sequences of cel-pri-mir-72, cel-pri-mir-64, cel-pri-mir-244 LS19, cel-pri-mir-244 LS16 (WT), and cel-pri-mir-36. The inserted nt are red letters. (**F–H**) Gels showing the results of the *in vitro* cleavage assays of WT and mutant cMPs on pri-miRNAs. (**I**) The cleavage accuracies of WT and mutant cMPs were calculated as the ratio of cleaved products at CL0 to the cleaved products at all the cleaved positions. The plots were generated from assays conducted in triplicate, as shown in (F–H). In all panels, the blue and green arrowheads indicate the cleavage sites of cMP (WT and mutants) at CL0, determined by cDrosha and Pasha, respectively. The gray arrowhead indicates the cleavage of cMP at other positions.

We predicted the structure of cDrosha using AlphaFold2 (46,47) and compared it with the published structures of hDROSHA/RNA (22–24). The predicted cDrosha and experimental hDROSHA structures showed a similar domain organization (Figure 5A). As hDROSHA uses the Belt helix and dsRBD to measure 13 bp from the basal junction to the cleavage sites (23,24), we wanted to investigate if these two domains also are important for the 16-bp measurement of cDrosha. We conducted HT cleavage assays for cMPΔBelt and cMPΔdsRBD (Figure 5B and Supplementary Figure S5A) and found these two mutant complexes only showed minor changes in cleavage efficiency and accuracy when compared with cMP-WT (Figure 5C, D), suggesting that the Belt helix and dsRBD in cDrosha are not essential for its substrate measurement. Next, we generated the cMPΔBeta complex by deleting the β-sheet (amino acids 502–512, Figure 5B and Supplementary Figure S5A), which is connected to the Belt helix and more distant from the catalytic center of cDrosha (Figure 5A). Interestingly, cMPΔBeta significantly reduced the cleavage efficiency and accuracy compared with cMP-WT (Figure 5C, D). These results suggest that the β-sheet region of cDrosha is critical for its ability to measure 16 bp from the basal junction, hence it is called β-16.

Next, we demonstrated that cMPΔBeta lost cleavage activity in cel-pri-mir-64, cel-pri-mir-86, cel-pri-mir-244 LS 19 and LS16 (WT), whose cleavage sites are mainly determined by cDrosha using its 16-bp measurement (Figure 5E–G, I). In contrast, cMPΔBelt and cMPΔdsRBD mutants cleaved these cel-pri-miRNAs similarly to cMP-WT (Figure 5E–G, I). In addition, cMP-WT and three cMP mutants similarly cleaved cel-pri-mir-36, whose cleavage is mainly determined by Pasha (Figure 5E, H, I). These results confirm the critical role of β-16 in the junction measurement and cleavage activity of cDrosha.

### Structural basis of Pasha measurement

**Figure 6.**
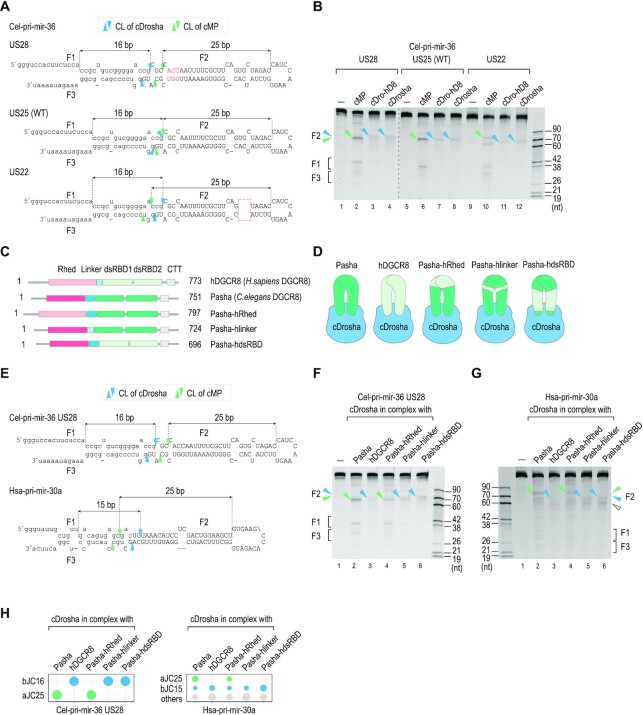
Structural basis of the Pasha measurement. (**A**) Diagrams and sequences of cel-pri-mir-36 and its variants. The inserted nt are red letters. The deleted nt are shown in the red dashed box. (**B**) Gel showing the results from the *in vitro* cleavage assays of cDrosha, cMP, and cDrosha-hDGCR8 (cDro-hD8). (**C**) The protein domains for hDGCR8 (hD8), Pasha, and mutant Pasha. Pasha-hRhed, Pasha-hlinker, and Pasha-hdsRBD contained the swapped Rhed, linker, and dsRBD from hDGCR8, respectively. The numbers indicate the positions of the amino acids in the polypeptides. Rhed, RNA-binding heme domain; linker, the connecting domain between Rhed and dsRBDs; dsRBD, double-stranded RNA-binding domain. (**D**) Schematics showing the five different complexes of cDrosha with Pasha, hDGCR8, Pasha-hRhed, Pasha-hlinker, or Pasha-hdsRBD. (**E**) Diagrams and sequences of cel-pri-mir-36 US28 and hsa-pri-mir-30a. (**F, G**) Gels showing the results of *in vitro* cleavage assays of WT and mutant cMPs (as shown in D) on the pri-miRNAs. (**H**) The cleavage accuracies of WT and mutant cMPs were calculated as the ratio of cleaved products at bJC16 (for cel-pri-mir-36 US28) or bJC15 (for hsa-pri-mir-30a), and aJC25 to the cleaved products at all positions. The plot was generated from the assays conducted in triplicate, as shown in (F, G). In all panels of Figure 6, the blue and green arrowheads indicate the cleavage sites of cMP (WT and mutants), determined by cDrosha and Pasha, respectively. The gray arrowhead indicates the cleavage of cMP at other positions.

The data in Figure 3 show that Pasha can cause cMP to cleave at different positions from the cleavage sites of cDrosha. This activity of Pasha seems to be different from hDGCR8, which only stimulates the cleavage sites predetermined by hDROSHA (21). We compared the effect of hDGCR8 and Pasha on the cleavage site choices of cDrosha in cel-pri-mir-36 US28, US25 and US22 variants. Consistent with the results in Figure 3F and G, Pasha changed the cleavage sites of cDrosha in the three cel-pri-mir-36 variants, resulting in different F2 fragments compared with cDrosha alone. In contrast, hDGCR8 did not alter the cleavage sites of cDrosha in the cDrosha-hDGCR8 complex, resulting in similar F2 fragments being generated as for cDrosha alone (Figure 6A, B). These results indicate that Pasha and hDGCR8 interact with and measure pri-miRNAs differently.

Next, we replaced the Rhed, dsRBD1-2, and linker of Pasha with the corresponding domain from hDGCR8, generating chimeric Pasha-hRhed, Pasha-hdsRBD, and Pasha-hlinker proteins (Figure 6C). We then purified three mutant cMP complexes containing cDrosha and each of these above chimeric proteins (Figure 6D, and Supplementary Figure S6A) and tested the cleavage sites of these complexes in the cel-pri-mir-36 US28. We found that with Pasha-hRhed, cDrosha still cleaved this cel-pri-miRNA variant using the 25-bp measuring mechanism, suggesting that the Rhed of Pasha is unnecessary for its 25-bp measurement (Figure 6E, F, H). However, Pasha-hdsRBD and Pasha-hlinker failed to change the cleavage sites of cDrosha (Figure 6E, F, H). Similar effects of these chimeric Pasha proteins on the cleavage sites of cDrosha were obtained with hsa-pri-mir-30a (Figure 6E, G, H). Thus, these data indicate that the ∼25-bp measuring mechanism of Pasha is dependent on its dsRBDs and linker region.

## DISCUSSION

**Figure 7.**
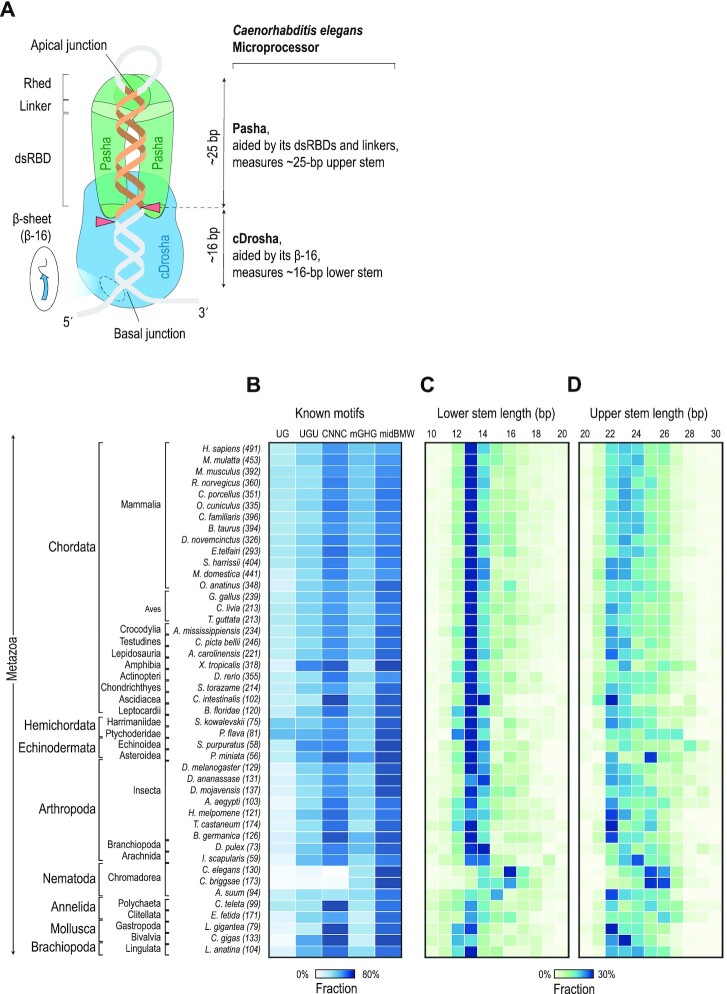
A model of *C. elegans* Microprocessor action on cel-pri-miRNAs. (**A**) cDrosha measures ∼16 bp of the lower stem of cel-pri-miRNAs and determines its cleavage sites. The cDrosha measurement is supported by the β-sheet located in its central domain. Pasha measures ∼25 bp of the upper stem of cel-pri-miRNAs and determines the cleavage sites of cDrosha in this location. The Pasha measurement is aided by its dsRBDs and linker region. These two measuring mechanisms, which are regulated by the two subunits, are coordinated for cMP to achieve optimal cleavage activity. (**B**) The percentage of pri-miRNAs containing UG, UGU, CNNC, mGHG or midBMW motifs in each of the 45 listed species. (C, D) The percentage of pri-miRNAs containing different lengths of (**C**) lower and (**D**) upper stem in each of the 45 species listed.

The first miRNA (lin-4) was discovered in *C. elegans* nearly 30 years ago (48,49). Eleven years after this initial discovery, MP was found in *C. elegans* and various other animals, including *D. melanogaster* and humans (13–16). Since then, due to the important role of miRNAs in various human diseases, the molecular mechanism of hMP has been intensively investigated. It is now well established that in the hMP complex, the pri-miRNA cleavage site is mainly determined by the ribonuclease, hDROSHA, which accurately measures (and cleaves) 13 bp from the basal junction of the stem-loop structure. The hDGCR8 component of hMP stimulates hDROSHA to cleave pri-miRNAs at this so-called productive site (i.e. 13-bp from the basal junction) and prevents it from cleaving at unproductive sites (i.e. 13-bp from the apical junction) and/or alternative sites (i.e. sites other than 13 bp from the basal junction) (3,21). hDGCR8 binds to the apical loop and a UGU motif, located ∼22–23 bp from the cleavage site. However, it does not possess a 22-bp measuring mechanism, and so it can only stimulate cleavage at the place predetermined by hDROSHA (18,21,31). Here, we reveal the molecular mechanism of *C. elegans* MP (cMP) and show that it is different from the human mechanism in several respects (Figure 7A). (1) cDrosha measures a longer distance (i.e. ∼16 bp) between the basal junction and its cleavage sites than hDROSHA (i.e. ∼13 bp). (2) Unlike hDGCR8, which does not have a measuring mechanism, Pasha measures ∼25 bp between the apical loop and the cleavage sites. In addition, Pasha works with cDrosha to determine the cleavage sites of cMP. (3) cDrosha and Pasha generate alternative cleavages when the two measuring mechanisms are incompatible with the same cleavage sites. In humans, however, alternative cleavages are mainly generated by hDROSHA alone (for example, hsa-pri-mir-342 (28,50,51)) or with support from the cofactor, SRSF3 (for example, hsa-pri-mir-142 (33)).

Our findings indicate that the efficiency of cMP cleavage in most cel-pri-miRNAs requires both cDrosha and Pasha but the accuracy of cleavage is dependent on either cDrosha or Pasha but usually not both. Therefore, cel-pri-miRNAs can be classified into three groups, cDrosha-dependent, Pasha-dependent, and cDrosha/Pasha-dependent cel-pri-miRNAs (Supplementary Figure S7A–C). In cDrosha-dependent cel-pri-miRNAs, the lower stem interacts strongly with cDrosha, whereas the upper stem binds only weakly to Pasha. In contrast, in Pasha-dependent cel-pri-miRNAs, the lower stem has a weak affinity with cDrosha, whereas the upper stem has a higher affinity with Pasha. With regards to cDrosha/Pasha-dependent cel-pri-miRNAs, the lower and upper stems interact strongly with cDrosha and Pasha, respectively.

Here, we present an atlas showing the cleavage sites of cMP (and its cDrosha component) in almost all the cel-pri-miRNAs. Consistent with the human atlas reported previously (33), we show that MPs often cleave pri-miRNAs at multiple sites. In addition, they cleave some pri-miRNAs at positions different from the annotated cleavage sites. Examples include cel-pri-mir-358 and cel-pri-mir-796 in this study, and hsa-pri-mir-152, hsa-pri-mir-342 described previously (33). This suggests that in human and *C. elegans* cells, MP might be supported by auxiliary factors to find the right cleavage sites.

The pri-miRNA structures of each species seem to have evolved to fit the measurement mechanisms of its MP. For example, pri-miRNAs from *Caenorhabditis briggsae* share similar structures to *C. elegans*, including an absence of the UG, UGU and CNNC motifs (Figure 7B), a longer lower stem of ∼16 bp (Figure 7C), and a longer upper stem of ∼25 bp (Figure 7D). In contrast, other animals possess pri-miRNAs that contain similar structures as humans, such as enriched UG, UGU and CNNC motifs, a shorter lower stem of ∼13 bp, and a shorter upper stem of ∼22 bp. This suggests that the MPs of *C. briggsae* and *C. elegans* have a similar mechanism, whereas those of other animals utilize similar mechanisms to hMP. It is also reported that Dicer-like proteins (DCL) in plants also contain a ∼15–17 bp measuring mechanism (52,53). This suggests that the longer lower stem measuring mechanism of cDrosha might be an ancient feature.

We also showed that the Belt helix region of cMP is not essential for the efficient and accurate cleavage of cel-pri-miRNAs unlike that of hMP. Indeed, in *C. elegans*, β-16 plays a more vital role in the cleavage activity of cDrosha. The Alphafold2-predicted structure of cDrosha shows that β-16 is more distant from the cleavage sites than the Belt helix, so it might in some way help cDrosha measure 16 bp instead of 13 bp. Previous studies of hMP (23,24) demonstrate the role of the Belt helix in the hDROSHA measuring mechanism; however, the involvement of β-16 in this measurement has yet to be tested. Perhaps, β-16 also contributes to the hDROSHA measurement by stabilizing or placing the Belt helix in the proper position. Although the Belt helix region of cDrosha is not directly involved in the measuring mechanism, it might still contribute in some way to the cleavage mechanism of the enzyme. This is because, as shown in previous human studies (23,24), the Belt helix possesses an RNA-binding affinity and is located near the lower stem. In addition, the HT and validated cleavage assay data we present here, show that in the absence of the dsRBD, the efficiency and accuracy of cMP cleavage are slightly affected. This is unlike the dsRBD in hMP, which is absolutely required for the efficient and accurate cleavage of many hsa-pri-miRNAs (1,27,35,36). We discovered that among the many cel-pri-miRNAs we tested, cMPΔdsRBD appeared to reduce the cleavage accuracy of cMP in cel-pri-mir-244 LS19 (Figure 5G) and cel-pri-mir-36 (Figure 5H). This suggests that the dsRBD of cMP might be important for the cleavage of some cel-pri-miRNAs that contain certain RNA features. Our study also indicates that the dsRBDs and linker region of Pasha are necessary for the 25-bp upper stem measurement. It would be of interest now, to solve the cMP/pri-miRNA structure to fully understand the structural basis of its substrate measurements.

## DATA AVAILABILITY

The DNA libraries were sequenced with Illumina NovaSeq 6000 system in 150 bp paired-end mode. Sequencing data were deposited in Gene Expression Omnibus (GEO, https://www.ncbi.nlm.nih.gov/geo/) with the accession number GSE212229.

## Supplementary Material

gkac1170_Supplemental_Files
