# Supplementary material for: Dissection of the Caenorhabditis elegans Microprocessor
Source: Nucleic Acids Res. 2023 Jan 4;51(4):1512–27. doi: 10.1093/nar/gkac1170 (PMC9976908; doi:10.1093/nar/gkac1170)
Supplement: gkac1170_Supplemental_Files [file gkac1170_supplemental_files.zip › Supplementary figures.pdf]

Supplementary Figure S1

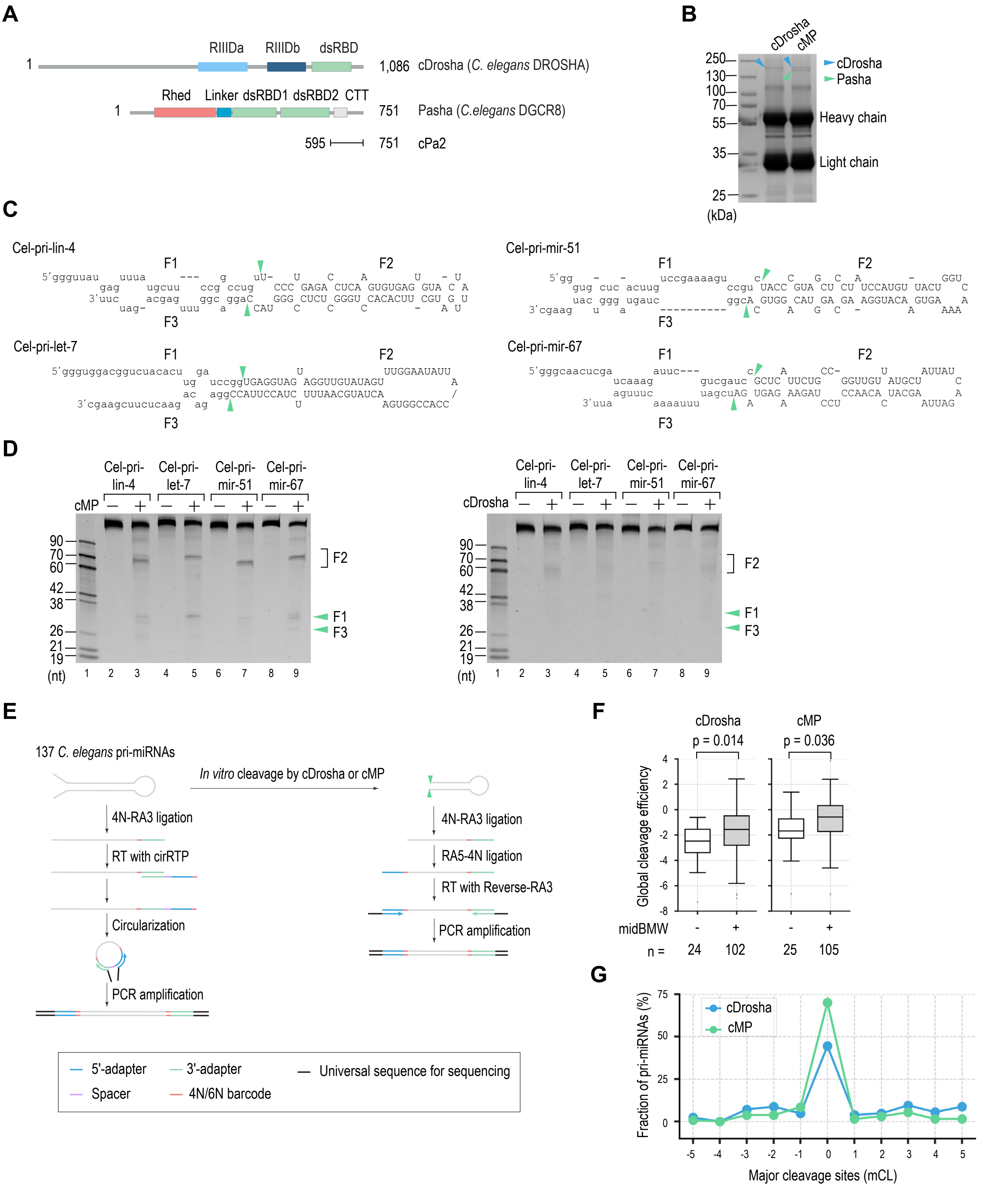

Supplementary Figure S1. High-throughput cleavage assays. (**A**) The protein domains of cDrosha and Pasha, two subunits of the Microprocessor complex in *C. elegans*, and the Pa2 fragment of Pasha. RIIIDa and RIIIDb are RNase III domains; dsRBD is double-stranded RNA binding domain; Rhed is RNA-binding heme domain; linker is the connecting domain between Rhed and dsRBDs. (**B**) The purified *C. elegans* Microprocessor (cMP, cDrosha-Pasha) and *C. elegans* DROSHA (cDrosha-NLScPa2) were assessed by SDS-PAGE. NLS is the nuclear localization sequence (PKKKRKV) and cPa2 is a fragment of Pasha at amino acids 595–751. The positions of cDrosha and Pasha as well as the heavy chain and light chain of the IgG used in the protein purification are indicated. (**C**) Diagrams and sequences of four representative *C. elegans* pri-miRNAs. The green arrowheads show the annotated cleavage sites in MirGeneDB. (**D**) Gels showing the results from *in vitro* cleavage assays of the four representative pri-miRNAs by cMP and cDrosha. (**E**) The cloning scheme of uncleaved substrates and cleaved F2 products from 137 *C. elegans* pri-miRNAs, as described in the Materials and Methods. 4N-RA3, 3'-adapter; RA5-4N, 5'-adapter; RT, reverse transcription; cirRTP, RT primer. (**F**) The global cleavage efficiency of cDrosha or cMP on the midBMW_none and midBMW cel-pri-miRNAs, respectively. The p-values were calculated by one-sided Wilcoxon rank-sum tests. (**G**) The fraction of cel-pri-miRNAs containing the major cleavage site at different positions. The cleavage accuracy score was estimated as the ratio of the product cleaved at each cleavage site to the total products cleaved at all positions. The major cleavage site (mCL) of a cel-pri-miRNA is its cleavage site (CL), with the highest cleavage accuracy score.

Supplementary Figure S2

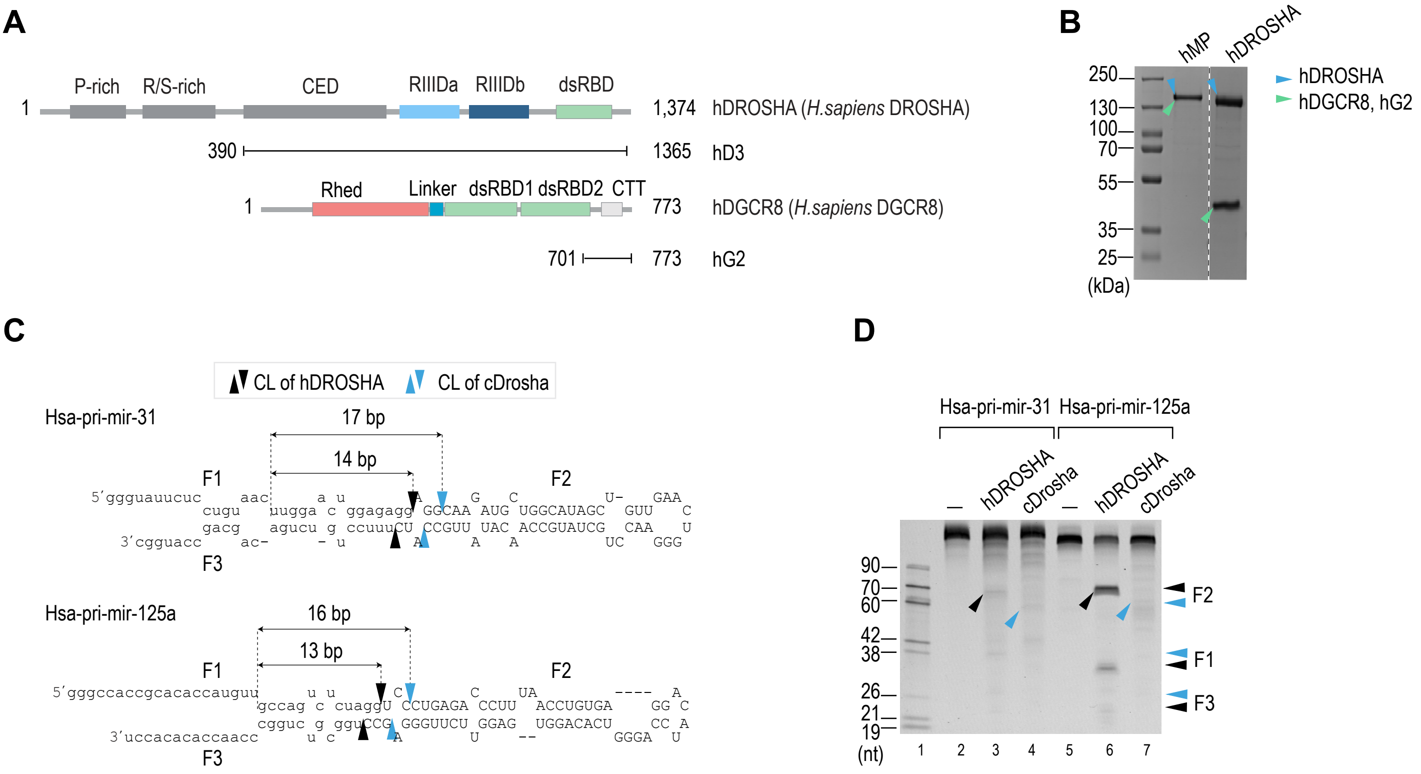

Supplementary Figure S2. cDrosha measures ~16 bp of the lower stem. (**A**) The protein domains of hDROSHA and hDGCR8, two subunits of the Microprocessor complex in humans and their fragments, hD3, and hG2. (**B**) The quality of human Microprocessor (hMP, NLShD3-hDGCR8) and human DROSHA (hD3-hG2) were assessed by SDS-PAGE. hD3 is a fragment of hDROSHA (amino acids 390–1365) and hG2 is a fragment of hDGCR8 (amino acids 701–773). The blue and green arrowheads indicate the position of hDROSHA, and hDGCR8 (or hG2), respectively. (**C**) Diagrams and sequences of two human pri-miRNAs, hsa-pri-mir-31, and hsa-pri-mir-125a. (**D**) Gel showing the results from *in vitro* cleavage assays of hDROSHA and cDrosha on human pri-miRNAs. The black and blue arrowheads indicate the cleavage sites of hDROSHA and cDrosha, respectively.

Supplementary Figure S3

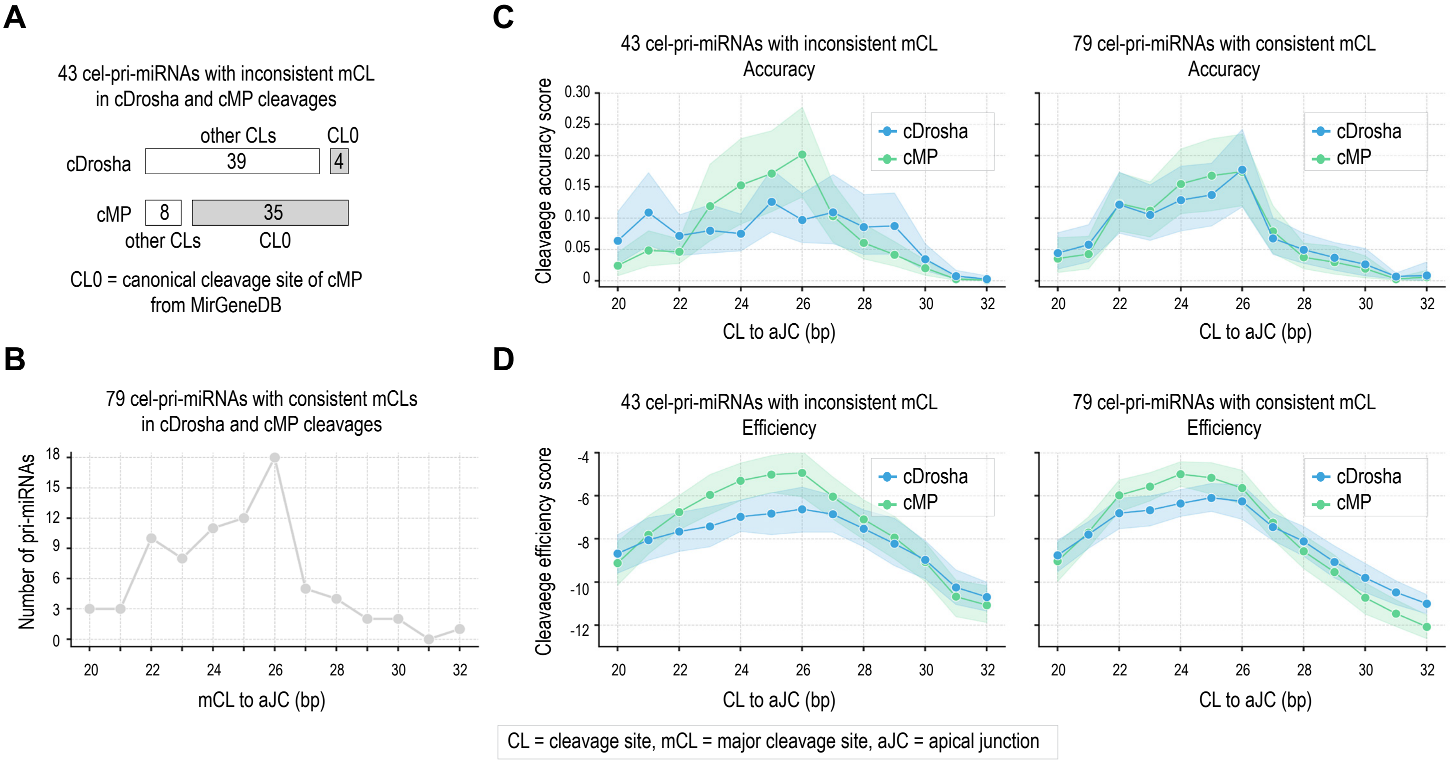

Supplementary Figure S3. Pasha measures ~25 bp of the upper stem. (**A**) The major cleavage sites of cDrosha and cMP in 43 cel-pri-miRNAs that had major cleavage sites distinct between the two, were classified as CL0 or non-CL0 (or other CLs). (**B**) The number of cel-pri-miRNAs containing consistent mCLs in both cDrosha and cMP cleavages at different distances to the apical junction (aJC). (**C**, **D**) The average (C) cleavage accuracy and (D) efficiency scores of cDrosha and cMP at different distances from the apical junction (aJC) for the two groups of cel-pri-miRNAs shown in Figure 3A. The cleavage accuracy and efficiency scores were estimated as the ratio of the products cleaved at each cleavage site to the total products cleaved at all positions and the ratio of the products cleaved at each cleavage site to the original substrate, respectively.

Supplementary Figure S4

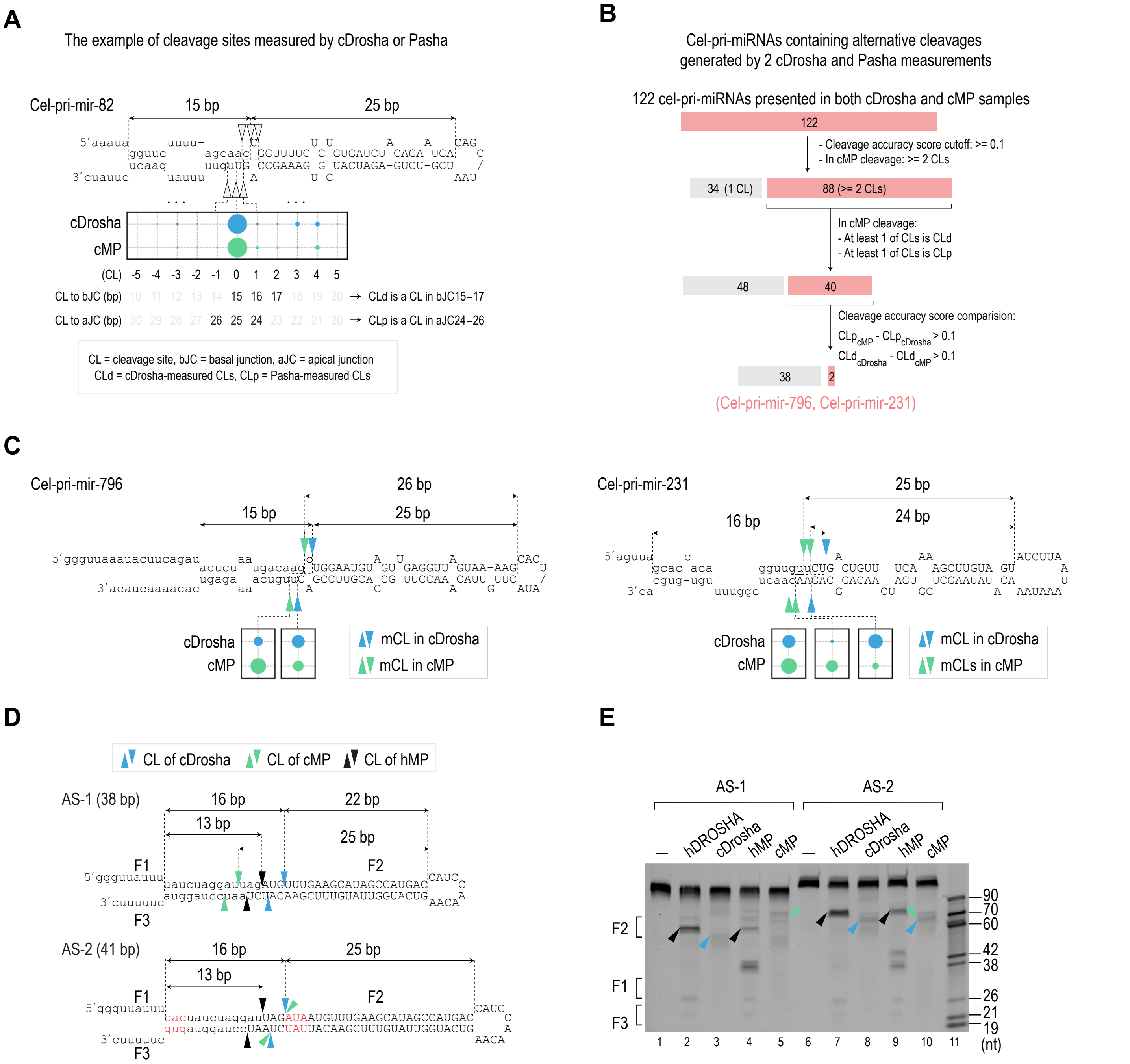

Supplementary Figure S4. The coordination of cDrosha and Pasha in determining the cleavage sites. (**A**) The example of cleavage sites (CL) measured by cDrosha (cDrosha-measured CL, CLd) or Pasha (Pasha-measured CL, CLp). CLd and CLp were defined as a CL in 15–17 bp from bJC (basal junction) and 24–26 bp from aJC (apical junction), respectively. (**B**) Identification of alternatively cleaved cel-pri-miRNAs by cDrosha-measured and Pasha-measured mechanisms in the HT cleavage assays. Among 122 cel-pri-miRNAs identified in cDrosha and cMP cleavage samples, we collected 88 cel-pri-miRNAs containing multiple CLs in the cMP cleavage sample, such that each CL had a cleavage accuracy score higher than 0.1. 40 of 88 cel-pri-miRNAs, containing at least one CLd and one CLp, were shortlisted. The CLp should increase its cleavage accuracy score in the cMP cleavage sample than the cDrosha cleavage sample. In contrast, the CLd should increase its cleavage accuracy score in the cDrosha cleavage sample than the cMP cleavage sample. Finally, 2 out of 40 cel-pri-miRNAs showed this expected increased CLp and CLd in the cMP cleavage and cDrosha cleavage samples, respectively ($CLp_{cMP} - CLp_{cDrosha} > 0.1$ and $CLd_{cDrosha} - CLd_{cMP} > 0.1$). (**C**) The cleavage patterns of cDrosha and cMP in cel-pri-mir-796 and cel-pri-mir-231. (**D**) Diagrams and sequences of artificial pri-miRNA substrates (AS-1 and AS-2). AS-1 comprised random nt sequences that did not map to any C. elegans miRNA loci, whereas AS-2 was modified from AS-1 by inserting 6 base pairs (highlighted in red) in the AS-1 stem. (**E**) Gel showing the results from the in vitro cleavage assays of hDrosha, cDrosha, hMP, and cMP for the AS-1 and -2 substrates. The black arrowhead indicate the cleavage site of hDROSHA and hMP. The blue and green arrowheads indicate the cleavage site of cMP, determined by cDrosha or Pasha, respectively.

Supplementary Figure S5

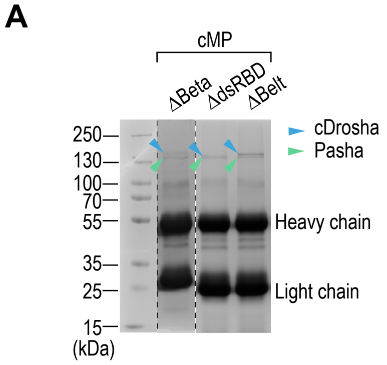

Supplementary Figure S5. Structural basis of cDrosha measurement. (**A**) The quality of mutant cMPs was assessed by SDS-PAGE. The positions of cDrosha and Pasha are indicated by the blue and green arrowheads, respectively. The positions of the heavy and light chains of the IgG used in the protein purification are indicated.

Supplementary Figure S6

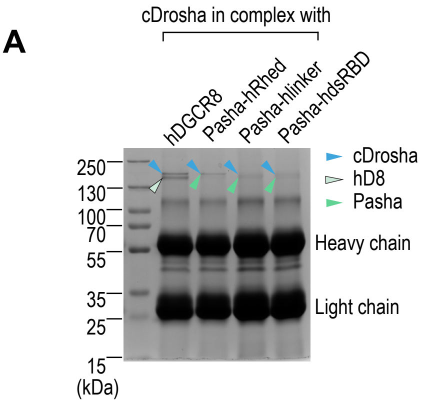

Supplementary Figure S6. Structural basis of Pasha measurement. (**A**) The quality of WT and mutant cMPs was assessed by SDS-PAGE. We coexpressed cDrosha containing protein G tag and hDGCR8 containing 10xHis tag. The coexpressed complex was purified sequentially by Ni-NTA resin and IgG beads. The coexistence of cDrosha and hDGCR8 after the final step of purification indicated that these two proteins interacted with each other. The positions of cDrosha, hD8, and Pasha are indicated by the blue, light green, and green arrowheads, respectively. The positions of heavy and light chains of the IgG used in protein purification are indicated.

Supplementary Figure S7

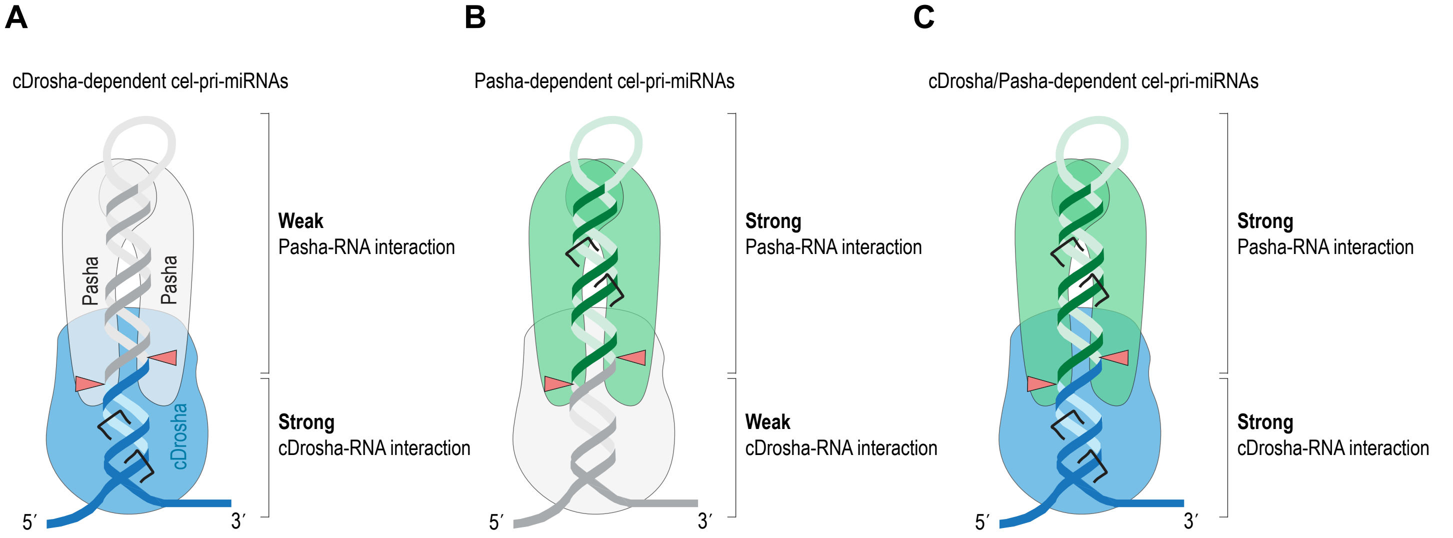

Supplementary Figure S7. Proposed models of cDrosha and/or Pasha-dependent pri-miRNAs. (**A**) cDrosha-dependent cel-pri-miRNAs. (**B**) Pasha-dependent cel-pri-miRNAs. (**C**) cDrosha/Pasha-dependent cel-pri-miRNAs. The red arrowheads indicate the cleavage sites of cDrosha. The black snaphooks indicate strong interaction.
